# Seroprevalence of hepatitis B virus and hepatitis C virus infection among Malaysian population

**DOI:** 10.1038/s41598-020-77813-5

**Published:** 2020-12-03

**Authors:** Nor Asiah Muhamad, Rimah Melati Ab.Ghani, Mohd Hatta Abdul Mutalip, Eida Nurhadzira Muhammad, Hasmah Mohamad Haris, Rozainanee Mohd Zain, Noraidatulakma Abdullah, Nor Azila Muhammad Azami, Nazihah Abd Jalal, Norliza Ismail, Nurul Ain Mhd Yusuf, Raihannah Othman, Azwa Shawani Kamalul Arifin, Mohd Shaharom Abdullah, Mohd Arman Kamaruddin, Muhammad Radzi Abu Hassan, Tahir Aris, Rahman Jamal

**Affiliations:** 1 Evidence-Based Healthcare Sector, National Institutes of Health, Ministry of Health Malaysia, 40170 Shah Alam, Malaysia; 2 Center for Communicable Diseases Epidemiology Research, Institute for Public Health, National Institutes of Health, Ministry of Health Malaysia, 40170 Shah Alam, Malaysia; 3 Institute for Medical Research, National Institutes of Health, Ministry of Health Malaysia, 40170 Shah Alam, Malaysia; 4 UKM Medical Molecular Biology Institute (UMBI), Universiti Kebangsaan Malaysia, Kuala Lumpur, Malaysia; 5 Hospital Sultanah Bahiyah, 05460 Alor Setar, Kedah Malaysia

**Keywords:** Immunology, Molecular biology, Diseases, Health care, Medical research, Molecular medicine, Risk factors

## Abstract

Malaysia is a country with an intermediate endemicity for hepatitis B. As the country moves toward hepatitis B and C elimination, population-based estimates are necessary to understand the burden of hepatitis B and C for evidence-based policy-making. Hence, this study aims to estimate the prevalence of hepatitis B and C in Malaysia. A total of 1458 participants were randomly selected from The Malaysian Cohort (TMC) aged 35 to 70 years between 2006 and 2012. All blood samples were tested for hepatitis B and C markers including hepatitis B surface antigen (HBsAg), anti-hepatitis B core antibody (anti-HBc), antibodies against hepatitis C virus (anti-HCV). Those reactive for hepatitis C were further tested for HCV RNA genotyping. The sociodemographic characteristics and comorbidities were used to evaluate their associated risk factors. Descriptive analysis and multivariable analysis were done using Stata 14. From the samples tested, 4% were positive for HBsAg (95% CI 2.7–4.7), 20% were positive for anti-HBc (95% CI 17.6–21.9) and 0.3% were positive for anti-HCV (95% CI 0.1–0.7). Two of the five participants who were reactive for anti-HCV had the HCV genotype 1a and 3a. The seroprevalence of HBV and HCV infection in Malaysia is low and intermediate, respectively. This population-based study could facilitate the planning and evaluation of the hepatitis B and C control program in Malaysia.

## Introduction

Hepatitis B virus (HBV) is a partially double stranded DNA virus that belongs to the hepatitis DNA viruses (hepadnaviruses), while hepatitis C virus (HCV) is a plus-stranded RNA virus of the *Flaviviridae* family. Infection with HBV and/or HCV were associated with the development of liver cirrhosis and hepatocellular carcinoma^1,2^. The World Health Organization (WHO) estimates that by 2017, approximately 248 million people will be living with chronic HBV infection and 110 million people with HCV infection; of these, 80 million people will have an active viral infection^3,4^. Most HBV and HCV infections are asymptomatic, and about 90% of patients infected with HCV are unaware that they have the virus that makes them a healthy carrier^5^. Nonetheless, both HBV and HCV infections are generally preventable, treatable and potentially curable if they are diagnosed at an early stage^1,2^.

The Ministry of Health (MoH), Malaysia has reported that about 5% of Malaysian population is infected with HBV^6^. Despite the incidence of HBV in Malaysia being constant between 11 and 15% for the past 5 years (since 2015) and the introduction of HBV vaccination in 1989, the incidence of hepatitis B (HB) is projected to increase between 2010 and 2040^7^. This will add an additional burden on health services due to the chronic sequelae of HB due to the higher rates of hospitalization and treatment for liver cirrhosis and hepatocellular carcinoma, which is currently among the eight most common types of cancer in Malaysia^8^. A population based seroprevalence study could provide insights on the current situation of HB in Malaysia including the impact of HB vaccination policy and existing screening strategies to further mitigate HB transmission in the community.

About 2 to 2.5% of the Malaysian population is infected with HCV^6^. The HCV infection in Malaysia is projected to rise steeply over the coming decades. A total of 64,000 hepatitis C-related death was projected to happen in 2039 with a total of 2002 and 540 individuals will developed decompensated cirrhosis and hepatocellular carcinoma, respectively^9^. Thus, it is important to determine the true prevalence of HBV and HCV infection in the Malaysian population in order to determine the burden of infection and to estimate the size of the chronically infected community and those in need of treatment. Such data can be used in health planning or policy decision making in order to reduce the transmission of HBV and HCV and the mortality rates. The data may also facilitate the health authorities to review priorities, improve and extend early screening and strategies to reduce the burden of chronic HBV and HCV infection. To date, no population-based studies have been conducted to ascertain the prevalence of HBV and HCV infections among the adult population of Malaysia.

Therefore, this study aims to determine the prevalence of HBV and HCV (seroprevalence) infection in Malaysian adult population by determining the presence of hepatitis B surface antigen (HBsAg), antibody to hepatitis B core antigen (anti-HBc) and antibody to hepatitis C virus (anti-HCV) in the serum.

## Results

The baseline characteristics for the 1458 The Malaysian Cohort (TMC) participants are presented in Supplementary Table S1. The cohort comprises randomly chosen individuals who were recruited between 2007 and 2012 and mostly in 2011 (24%). Most of the participants were between 45 and 54 years old, Malays (40%), married (89%), had attained secondary education level (51%), working in the non-governmental sectors (43%), from the state of Selangor (24%) and urban area (71%).

Majority of the participants had no history of hepatitis A and B immunisation (82%), no history of chronic hepatitis (99.8%) and no history of blood transfusion (92%). The proportion was almost similar for individuals with or without history of surgery. All the participants had no family history of chronic hepatitis.

### Hepatitis B surface antigen (HBsAg)

The seroprevalence of HBsAg was 4% as depicted in Table 1. There were differences between patients’ characteristics and status of HBsAg test. Those were positive HBsAg were aged between 55 and 64 years old (5%), male (5%), from the rural area (5%), among the Bumiputera ethnic group in Sabah (11%), married (4%), with no formal education (8%), unemployed (4%) and with no history of blood transfusion (3%). It is interesting to know that although no history of hepatitis immunization was statistically associated with HBsAg positivity, for immunized persons we found the highest proportion of positives in those vaccinated against hepatitis A virus. In addition, those who had history of chronic hepatitis was statistically associated with HBsAg positivity. However, only gender, ethnicity and history of chronic hepatitis disease showed significant differences with the status of HBsAg test. There were four out of 1458 samples which were equivocal. All equivocal samples were Malays, had no history of chronic hepatitis and blood transfusion, were married and working with the non-government sector.

**Table 1:** Distribution of hepatitis B surface antigen (HBsAg) in Malaysian adult population (n = 1458).

Characteristics	n (%)	Hepatitis B surface antigen, n (%)			p
		Negative	Equivocal	Positive	
Total	1458 (100)	1401 (96)	4 (0)	53 (4)	
		[95–97]	[0.1–0.5]	[3–5]	
**Year of recruitment**					
2007	28 (2)	27 (96)	0 (0)	1 (4)	0.939
2008	161 (11)	155 (96)	1 (1)	5 (3)	
2009	273 (19)	263 (96)	1 (1)	9 (3)	
2010	308 (21)	296 (96)	0 (0)	12 (4)	
2011	351 (24)	335 (95)	2 (1)	14 (4)	
2012	337 (23)	325 (96)	0 (0)	12 (4)	
**Age group**					
≤ 44	399 (27)	386 (97)	1 (0)	12 (3)	0.324
45–54	624 (43)	603 (97)	3 (0)	18 (3)	
55–64	415 (28)	393 (95)	0 (0)	22 (5)	
65–70	20 (1)	19 (95)	0 (0)	1 (5)	
**Gender**					
Male	718 (49)	680 (94)	4 (1)	34 (5)	0.01*
Female	740 (51)	721 (97)	0 (0)	19 (3)	
**Ethnicity**					
Malay	588 (40)	574 (98)	2 (0)	12 (2)	< 0.001*
Chinese	513 (35)	484 (94)	2 (1)	27 (5)	
Indians	180 (12)	180 (100)	0 (0)	0 (0)	
Bumiputera Sabah	93 (6)	83 (89)	0 (0)	10 (11)	
Bumiputera Sarawak	53 (4)	49 (92)	0 (0)	4 (8)	
Others	31 (2)	31 (100)	0 (0)	0 (0)	
**Marital status**					
Single	73 (5)	71 (97)	0 (0)	2 (3)	0.965
Married	1300 (89)	1246 (96)	4 (0)	50 (4)	
Widow/widower	62 (4)	61 (98)	0 (0)	1 (2)	
Separated	5 (1)	5 (100)	0 (0)	0 (0)	
Divorced	18 (1)	18 (100)	0 (0)	0 (0)	
**Education level**					
No formal education	25 (2)	23 (92)	0 (0)	2 (8)	0.533
Primary	417 (29)	397 (95)	1 (0)	19 (5)	
Secondary	742 (51)	713 (96)	2 (0)	27 (4)	
Tertiary	274 (19)	268 (98)	1 (0)	5 (2)	
**Occupation**					
Unemployed	500 (34)	481 (96)	0 (0)	19 (4)	0.486
Non-government	628 (43)	601 (96)	4 (0)	23 (4)	
Government	248 (17)	240 (97)	0 (0)	8 (3)	
Self-employed	82 (6)	79 (96)	0 (0)	3 (4)	
**Locality**					
Urban	1035 (71)	1001 (97)	3 (0)	31 (3)	0.123
Rural	423 (29)	400 (95)	1 (0)	22 (5)	
**History of immunisation**					
No	1198 (82)	1147 (96)	4 (0)	47 (4)	0.747
Hepatitis A	41 (3)	39 (95)	0 (0)	2 (5)	
Hepatitis B	180 (12)	177 (98)	0 (0)	3 (2)	
Hepatitis A and hepatitis B	39 (3)	38 (97)	0 (0)	1 (3)	
**History of chronic hepatitis disease**					
Yes	2 (1)	0 (0)	0 (0)	2 (100)	< 0.001*
No	1456 (99)	1401 (96)	4 (0)	51 (4)	
**Family history of chronic hepatitis**					
Yes	0 (0)	0 (0)	0 (0)	0 (0)	
No	1458 (100)	1401 (96)	4 (0)	53 (4)	
**History of surgery**					
Yes	794(54)	762 (96)	2 (0)	30 (4)	0.936
No	664 (46)	639 (96)	2 (0)	23 (4)	
**History of blood transfusion**					
Yes	117 (8)	110 (94)	0 (0)	7 (6)	0.311
No	1341 (92)	1291 (96)	4 (0)	46 (4)	

*Indicate significant differences, p < 0.05.

Gender, ethnicity and locality were significantly associated with HBsAg seropositivity in the crude analysis (Table 2). The final multivariable models for HBsAg seropositivity included gender, ethnicity, locality, and immunisation history, history of surgery and history of blood transfusion. All these factors contributed to 8% risk of HBsAg seropositivity. History of chronic hepatitis was removed from the model due to high collinearity. Immunisation history, history of surgery and history of blood transfusion were included in the final models due to the clinical importance of the information. None of the other risk factors showed association with the status of HBsAg (p < 0.20) or were retained in the multivariable model.

**Table 2:** Sociodemographic factors associated with positivity of hepatitis B surface antigen (HBsAg) in Malaysian adult population.

Characteristics	Crude OR (95% CI)	p	Adjusted OR (95% CI)	p	R^2^
**Gender**					
Female (Ref)	1		1		
Male	1.9 (1.1, 3.3)	0.029*	2.0 (1.1, 3.8)	0.021*	8%
**Ethnicity**					
Malay (Ref)	1		1		
Chinese	2.7 (1.3, 5.3)	0.005*	4.9 (2.2, 11.2)	< 0.001*	
Bumiputera Sabah	5.8 (2.4, 13.8)	< 0.001*	4.6 (1.9, 11.2)	0.001*	
Bumiputera Sarawak	3.9 (1.2, 12.6)	0.022*	3.9 (1.2, 12.8)	0.027*	
**Locality**					
Urban (Ref)	1		1		
Rural	1.8 (1.0, 3.1)	0.044*	2.1 (1.0, 4.3)	0.044*	
**History of immunisation**					
No (Ref)	1		1		
Hepatitis A	1.3 (0.3, 5.4)	0.758	1.3 (0.3, 5.5)	0.769	
Hepatitis B	0.4 (0.1, 1.4)	0.143	0.3 (0.1, 1.2)	0.082	
Hepatitis A and B	0.6 (0.1, 4.8)	0.668	0.5 (0.1, 3.9)	0.507	
**History of surgery**					
No (Ref)	1		1		
Yes	1.1 (0.6, 1.9)	0.749	1.3 (0.7, 2.3)	0.456	
**History of blood transfusion**					
No (Ref)	1		1		
Yes	1.8 (0.8, 4.1)	0.163	2.4 (0.9, 5.9)	0.054	

*Indicate significant differences, p < 0.05.

In the final model, after adjusting for other confounding factors, males had two times significantly higher odds of HBsAg seropositivity compared to females (OR 2.0, 95% CI 1.1–3.8, p = 0.02). Compared to the Malays, the Chinese had the significantly highest odds in HBsAg seropositivity (OR 4.9, 95% CI 2.2–11.2, p < 0.001), followed by Bumiputera Sabah (OR 4.6, 95% CI 1.9–11.2, p = 0.001) and Bumiputera Sarawak (OR 3.9, 95% CI 1.2–12.8, p = 0.027). Those who lived in rural areas had a twofold significantly higher risk of HBsAg seropositivity than those who lived in urban areas (OR 2.1, 95% CI 1.0–4.3, p = 0.044). Participants who had a history of blood transfusion had higher risk than those who did not have history of blood transfusion, although the result was significant (OR 2.4, 95% CI 0.9–5.9, p = 0.054). Even though the results were not significant, the individuals with history of surgery and history of hepatitis A immunisation showed 26% and 25% higher HBsAg seropositivity respectively.

### Antibody to hepatitis B core antigen (anti-HBc)

The prevalence of hepatitis B core antigen (anti-HBc) positive was 20% as shown in Table 3. Majority of those with hepatitis B core antigen seropositivity (anti-HBc) were recruited in 2007 (25%), aged 65 to 70 (35%), and working in non-government sectors (20%). Similar to HBsAg, those were male (23%) and lived in rural areas (23%), were Bumiputera Sabah (44%), had no formal education (40%), with history of chronic hepatitis, were more likely to be positive in hepatitis B core antigen serology. However, only age, gender, ethnicity, education level, state, locality and history of chronic hepatitis were significantly associated with the status of anti-HBC serology. There were 53 (4%) participants who were positive for both HBsAg and anti-HBc.

**Table 3:** Distribution of antibody to hepatitis B core antigen (anti-HBc) in Malaysian adult population (n = 1458).

Characteristics	n (%)	Hepatitis B core antigen, n (%)			p
		Negative	Equivocal	Positive	
Total	1458 (100)	1049 (72)	119 (8)	290 (20)	
		[68.9–74.3]	[6.8–9.5]	[17.6–21.9]	
**Year of recruitment**					
2007	28 (2)	21 (75)	0 (0)	7 (25)	0.108
2008	161 (11)	113 (70)	10 (6)	38 (24)	
2009	273 (19)	192 (70)	26 (10)	55 (20)	
2010	308 (21)	217 (70)	30 (10)	61 (20)	
2011	351 (24)	244 (70)	26 (7)	81 (23)	
2012	337 (23)	262 (78)	27 (8)	48 (14)	
**Age group**					
≤ 44	399 (27)	325 (81)	19 (5)	55 (14)	< 0.001*
45–54	624 (43)	447 (72)	51 (8)	126 (20)	
55–64	415 (28)	267 (64)	46 (11)	102 (25)	
65–70	20 (1)	10 (50)	3 (15)	7 (35)	
**Gender**					
Male	718 (49)	485 (68)	65 (9)	168 (23)	0.001*
Female	740 (51)	564 (76)	54 (7)	122 (17)	
**Ethnicity**					
Malay	588 (40)	445 (77)	49 (8)	94 (16)	< 0.001*
Chinese	513 (35)	343 (67)	41 (8)	129 (25)	
Indians	180 (12)	167 (93)	5 (3)	8 (4)	
Bumiputera Sabah	93 (6)	43 (46)	9 (10)	41 (44)	
Bumiputera Sarawak	53 (4)	25 (47)	12 (23)	16 (30)	
Others	31 (2)	26 (84)	3 (10)	2 (6)	
**Marital status**					
Single	73 (5)	59 (81)	3 (4)	11 (15)	0.504
Married	1300 (89)	927 (71)	112 (9)	261 (20)	
Widow/widower	62 (4)	45 (73)	3 (5)	14 (23)	
Separated	5 (0)	3 (60)	0 (0)	2 (40)	
Divorced	18 (1)	15 (83)	1 (6)	2 (11)	
**Education level**					
No formal education	25 (2)	12 (48)	3 (12)	10 (40)	< 0.001*
Primary	417 (29)	269 (65)	45 (11)	103 (25)	
Secondary	742 (51)	558 (75)	53 (7)	131 (18)	
Tertiary	274 (19)	210 (77)	18 (7)	46 (17)	
**Occupation**					
Unemployed	500 (34)	359 (72)	42 (8)	99 (20)	0.328
Non-government	628 (43)	459 (73)	42 (7)	127 (20)	
Government	248 (17)	177 (71)	23 (9)	48 (19)	
Self-employed	82 (6)	54 (64)	12 (15)	16 (20)	
**Locality**					
Urban	1035 (71)	775 (75)	66 (6)	194 (19)	< 0.001*
Rural	423 (29)	274 (65)	53 (12)	96 (23)	
**History of immunisation**					
No	1198 (82)	844 (70)	103 (9)	251 (21)	0.052
Hepatitis A	41 (3)	30 (73)	4 (10)	7 (17)	
Hepatitis B	180 (12)	139 (77)	12 (7)	29 (16)	
Hepatitis A and hepatitis B	39 (3)	36 (92)	0 (0)	3 (8)	
**History of chronic hepatitis disease**					
Yes	2 (1)	0 (0)	0 (0)	2 (100)	0.018*
No	1456 (99)	1049 (72)	119 (8)	288 (20)	
**Family history of hepatitis**					
Yes	0 (0)	0 (0)	0 (0)	0 (0)	
No	1458 (100)	1049 (72)	119 (8)	290 (20)	
**History of surgery**					
Yes	794 (54)	569 (72)	68 (8)	157 (20)	0.828
No	664 (46)	480 (72)	51 (8)	133 (20)	
**History of blood transfusion**					
Yes	117 (8)	80 (68)	10 (9)	27 (23)	0.638
No	1341 (92)	969 (72)	109 (8)	263 (20)	

*Indicate significant differences, p < 0.05.

The final multivariable model consisted of age, gender, ethnicity, state, history of immunisation, history of surgery and history of blood transfusion, and together these factors contributed to 10% of anti-HBc seropositivity (Table 4). The risk of anti-HBc seropositivity significantly increased with age and was 53% higher among males than females. The risk was significantly higher among Bumiputera Sabah, followed by Chinese and Bumiputera Sarawak. However, anti-HBc seropositivity was substantially higher by almost threefold in those from the state of Terengganu. Those who had history of hepatitis B immunisation only and those who had both A and B immunisation were significantly protected against HBV compared to those who had no history of immunisation.

**Table 4:** Sociodemographic factors associated with positivity of hepatitis B core antigen (anti-HBc) in Malaysian adult population.

Characteristics	Crude OR (95% CI)	p	Adjusted OR (95% CI)	p	R^2^
**Age category**					9.51
35–45	1		1		
45–54	1.6 (1.1, 2.2)	0.009*	1.6 (1.1, 2.3)	0.015*	
55–64	2.0 (1.4, 2.9)	< 0.001*	1.9 (1.3, 2.9)	0.001*	
> 65	3.4 (1.3, 8.8)	0.013*	2.7 (1.0, 7.4)	0.059	
**Gender**					
Female	1		1		
Male	1.6 (1.2, 2.0)	0.001*	1.5 (1.2, 2.0)	0.003*	
**Ethnicity**					
Malay	1		1		
Chinese	1.8 (1.3, 2.4)	< 0.001*	2.2 (1.6, 3.2)	< 0.001*	
Indian	0.2 (0.1, 0.5)	< 0.001*	0.3 (0.1, 0.6)	0.001	
Others	0.4 (0.1, 1.5)	0.17	0.4 (0.1, 1.8)	0.242	
Bumiputera Sabah	4.1 (2.6, 6.6)	< 0.001*	4.7 (2.0, 11.2)	< 0.001*	
Bumiputera Sarawak	2.3 (1.2, 4.3)	0.01*	2.1 (0.9, 5.2)	0.108	
**State**					
Johor (Ref)	1		1		
Kedah	0.5 (0.2, 1.3)	0.186	0.8 (0.3, 2.0)	0.602	
Kelantan	1.0 (0.4, 2.3	0.905	1.3 (0.5, 3.2)	0.603	
Melaka	0.9 (0.2, 3.3)	0.86	1.0 (0.2, 3.9)	0.997	
Negeri Sembilan	0.8 (0.3, 1.8)	0.534	1.0 (0.4, 2.5)	0.982	
Pahang	1.4 (0.7, 2.9)	0.335	1.6 (0.8, 3.2)	0.232	
Perak	1.0 (0.6, 2.0)	0.899	1.3 (0.7, 2.6)	0.389	
Pulau Pinang	0.4 (0.1, 1.3)	0.14	0.4 (0.1, 1.4)	0.147	
Sabah	2.6 (1.5, 4.5)	0.001*	1.1 (0.5, 2.7)	0.767	
Sarawak	1.6 (0.9, 3.0)	0.118	1.4 (0.6, 3.0)	0.452	
Selangor	0.9 (0.5, 1.6)	0.774	1.2 (0.7, 2.0)	0.629	
Terengganu	2.3 (1.0, 5.1)	0.043*	3.0 (1.3, 6.9)	0.01*	
WP Kuala Lumpur	1.2 (0.7, 2.1)	0.544	1.1 (0.6, 2.0)	0.71	
**History of immunisation**					
No (Ref)	1		1		
Hepatitis A	0.8 (0.3, 1.8)	0.549	0.7 (0.3, 1.6)	0.394	
Hepatitis B	0.7 (0.5, 1.1)	0.134	0.6 (0.4, 1.0)	0.041*	
Hepatitis A and B	0.3 (0.1, 1.0)	0.056	0.3 (0.1, 1.0)	0.045*	
**History of surgery**					
No (Ref)	1		1		
Yes	1.0 (0.8, 1.3)	0.903	1.0 (0.7, 1.3)	0.862	
**History of blood transfusion**					
No (Ref)	1		1		
Yes	1.2 (0.8, 1.9)	0.369	1.5 (0.9, 2.5)	0.133	

*Indicate significant differences, p < 0.05.

### Antibody to hepatitis C virus (anti-HCV)

The hepatitis C seropositivity (anti-HCV) was 0.3% (Table 5). Majority of those who were anti-HCV positive were recruited in 2011, aged between 55 and 64 years old, males, Malays, single, had primary education, self-employed, had no history of immunisation, no history of chronic hepatitis, had history of surgery and had history of blood transfusion. However, none of the risk factors were significantly associated with the serology status of anti-HCV. Interestingly, two of them (0.2%) were positive for both anti-HCV and anti-HBc.

**Table 5:** Distribution of antibody to hepatitis C virus (anti-HCV) in Malaysian adult population (n = 1457).

Characteristics	n (%)	Hepatitis C virus, n (%)			p
		Negative	Equivocal	Positive	
Total	1458 (100)	1452 (99)	1 (0)	5 (1)	
		[99.2–99.9]	[0.0–0.2]	[0.1–0.7]	
**Year of recruitment**					
2007	28 (2)	28 (100)	0 (0)	0 (0)	0.895
2008	161 (11)	161 (100)	0 (0)	0 (0)	
2009	273 (19)	272 (100)	0 (0)	1 (0)	
2010	308 (21)	306 (100)	1 (0)	1 (0)	
2011	351 (24)	349 (99)	0 (0)	2 (1)	
2012	337 (23)	336 (100)	0 (0)	1 (0)	
**Age group**					
≤ 44	399 (27)	398 (99)	0 (0)	1 (1)	0.941
45–54	624 (43)	621 (99)	1 (0)	2 (0)	
55–64	415 (28)	413 (99)	0 (0)	2 (0)	
65–70	20 (2)	20 (100)	0 (0)	0 (0)	
**Gender**					
Male	718 (49)	714 (99)	1 (0)	3 (1)	0.531
Female	740 (51)	738 (99)	0 (0)	2 (1)	
**Ethnicity**					
Malay	588 (40)	585 (99)	0 (0)	3 (0.51)	0.334
Chinese	513 (35)	511 (100)	1 (0)	1 (0)	
Indians	180 (12)	180 (100)	0 (0)	0 (0)	
Bumiputera Sabah	93 (6)	93 (100)	0 (0)	0 (0)	
Bumiputera Sarawak	53 (4)	53 (100)	0 (0)	0 (0)	
Others	31 (2)	30 (97)	0 (0)	1 (3)	
**Marital status**					
Single	73(5)	71 (97)	0 (0)	2 (3)	0.106
Married	1300 (89)	1296 (99)	1 (1)	3 (0)	
Widow/widower	62 (4)	62 (100)	0 (0)	0 (0)	
Separated	5 (0)	5 (100)	0 (0)	0 (0)	
Divorced	18 (1)	18 (100)	0 (0)	0 (0)	
**Education level**					
No formal education	25 (2)	25 (100)	0 (0)	0 (0)	0.966
Primary	417 (29)	415 (99)	0 (0)	2 (1)	
Secondary	742 (51)	739 (99)	1 (0)	2 (2)	
Tertiary	274 (19)	273 (99)	0 (0)	1 (1)	
**Occupation**					
Unemployed	500 (34)	498 (99)	0 (0)	2 (1)	0.666
Non-government	628 (43)	625 (99)	1 (0)	2 (1)	
Government	248 (17)	248 (100)	0 (0)	0 (0)	
Self-employed	82 (6)	81 (99)	0 (0)	1 (1)	
**Locality**					
Urban	1035 (71)	1033 (99)	0 (0)	2 (1)	0.091
Rural	423 (29)	419 (99)	1 (0)	3 (1)	
**History of immunisation**					
No	1198 (82.17)	1193 (99.58)	0 (0.00)	5 (0.42)	0.225
Hepatitis A	41 (2.81)	41 (100.00)	0 (0.00)	0 (0.00)	
Hepatitis B	180 (12.35)	179 (99.44)	1 (0.56)	0 (0.00)	
Hepatitis A and hepatitis B	39 (2.67)	39 (100.00)	0 (0.00)	0 (0.00)	
**History of chronic hepatitis**					
Yes	2 (1)	2 (100)	0 (0)	0 (0)	0.996
No	1456 (99)	1450 (99)	1 (0)	5 (1)	
**Family history of chronic hepatitis**					
Yes	0 (0)	0 (0)	0 (0)	0 (0)	
No	1458 (100)	1452 (99)	1 (0)	5 (1)	
**History of surgery**					
Yes	794 (54)	788 (99)	1 (0)	5 (1)	0.081
No	664 (46)	664 (100)	0 (0)	0 (0)	
**History of blood transfusion**					
Yes	117 (8)	116 (99)	0 (0)	1 (1)	0.588
No	1341 (92)	1336 (99)	1 (0)	4 (1)	

*Indicate significant differences, p < 0.05.

The final multivariable model included marital status and history of blood transfusion which contributed to 13% of anti-HCV seropositivity (Table 6). Those who were married had lower risk of getting anti-HCV seropositivity compared to those who were single. While those who had history of blood transfusion had two times higher risk of getting anti-HCV seropositivity compared to those who had no history.

**Table 6:** Sociodemographic factors associated with positivity of hepatitis C virus antibodies (anti-HCV) in Malaysian adult population.

Characteristics	Crude OR (95% CI)	p	Adjusted OR (95% CI)	p	R^2^
**Marital status**					
Single	1		1		
Married	0.1 (0.1, 0.5)	0.007*	0.1 (0.1, 0.3)	0.001*	13%
**History of blood transfusion**					
No	1		1		
Yes	2.9 (0.3, 26.0)	0.346	2.0 (0.2, 19.8)	0.548	

*Indicate significant differences, p < 0.05.

## Discussion

This study showed that the prevalence of HBsAg and anti-HBc among the Malaysian adult population were 4% and 20%, respectively. The prevalence of HBsAg positive was higher than the previous studies conducted among the blood donors in Kelantan (1%) and thalassaemic patients in the National University of Malaysia hospital (HUKM) (2%), but lower than previous studies conducted among Negrito tribe in Kelantan (9%) and Malaysian volunteers in 1997 (5%)^10–13^. In contrast, the prevalence of anti-HBc in Malaysia from this study was lower than the prevalence among the adult population in Turkey (23%), Romania (27%), Northeast China (36%) and Korea (39%)^14–17^. However, the prevalence of anti-HBc positive in Malaysia is higher than in Croatia (7%), France (7%), Germany (9%), and Iran (16%)^18–21^.

The low prevalence of HBsAg among our participants may suggest that less people were acutely infected at the moment of blood collection. Result from this study indicated that HB endemicity level in Malaysia is intermediate^13^. Our study showed that being male was significantly associated with HBsAg seropositivity and similar finding were reported in Brazil and China^22,23^. Our study also found that those who lived in the rural areas had a higher risk of being HBsAg positive. There are several factors that may be associated with higher prevalence of HBsAg positive in rural areas including limited access to health care services and low vaccination coverage^12,24^.

The high prevalence of anti-HBc could be from acquired immunity from natural infection because the HBV vaccination program in Malaysia was introduced in 1989 and all the participants were born before 1989^25^. This might explain the reason we found that the prevalence of anti-HBc was higher among older people and those who were not vaccinated in this study. This would imply that most of them were asymptomatic and without any treatment, might lead to the chronic sequalae of HB. These individuals, including their family members who are probably undiagnosed and asymptomatic, pose a potential risk for the mode of transmission for hepatitis. The similar finding were reported in Brazil, Germany, and Thailand where prevalence of anti-HBc were higher in those people who were born before the HB immunisation program was started^22,26,27^.

In this study, HBsAg and Anti-HBc positivity was found to be significantly associated with males. Several factors that may be attributed to high prevalence of HbsAg and Anti-HBc in men including high-risk sexual behavior and the usage of intravenous drug^27,28^. Our study also revealed that HB infection was prevalent in certain ethnic groups such as the Chinese, Bumiputera Sarawak and Bumiputera Sabah. Previous studies also reported that HBsAg positivity was more prevalent among the Chinese than other ethnic group in Malaysia^6,13^. Although the reasons behind the high rate of HB in certain ethnic groups are still unknown, previous study suggested that high-risk behaviour and cultural activity, such as unhygienic tattooing, body piercing, high-risk sexual activities, and alcohol consumption may have been contributed to high prevalence of HBsAg and Anti-HBc^22,29^. Thus, further study on the association of lifestyles, behaviour or occupational risk exposures and HBV infection in different populations and ethnicities were needed in order to explain the different levels of HB infection between different populations.

This study also found the prevalence of HCV was only 0.34%, which is a huge discrepancy as compared to the estimated prevalence based on modelling and routine screening in Malaysia^6,9,30^. This level was lower than in the previous studies conducted among blood donors in Malaysia in 1993 and 2012 and among adult population in Iran (0.5%), Oslo (0.7%), France (0.8%), Croatia (0.9%), Morocco (2%), Czech Republic (2%), and Romania (3%)^18,19,21,31–36^. HCV genotype 1a and 3a were found in the HCV-positive samples, and those two genotypes are the common HCV circulating genotypes in Malaysia^37,38^. Interestingly, in this study, there was no difference between most of the basic sociodemographic factors and HCV infection except for marital status. We found that being married was significantly associated with HCV infection. Similar finding was found in Pakistan whereby being married was significantly associated with higher odds of acquiring HCV infection and sexual transmission between spouses may increase the horizontal transmission between healthy individual and HCV-infected individual^39^.

This study has several limitations including the samples selection was based on simple random sampling and limited questionnaires to investigate further risk factors of hepatitis. Thus, it is recommended to conduct a serosurvey using proper randomization with the appropriate sampling design to obtain the true estimates of HBV and HCV in the population-based setting and incorporate questions that will examine a wider set of risk factors in order to obtain a better understanding of hepatitis infection while controlling for bias^22,40,41^. This is important to further understand the socio-economic impact of hepatitis infection thus enabling better management and specific intervention on the susceptible and vulnerable population.

As a conclusion, we found that the prevalence of HBsAg, anti-HBc and anti-HCV positivity among Malaysian adult population were 4%, 20%, and 0.3%, respectively. The prevalence of anti-HBc was high among older adults with low immunisation coverage and with no reported history of chronic HB. This indicate the importance of hepatitis screening and testing to prevent the transmission in the family and community. Awareness on hepatitis could also help to avoid the chronic sequelae if the infected person could seek early treatment. It is vital to identify social and behaviour risk factors of these susceptible populations who have not been vaccinated in order to understand how they acquire the infection.

## Methods

### Study population

The Malaysian Cohort (TMC) project is a prospective population-based study that was initiated in 2006^42^. The project has recruited 106,527 Malaysians aged 35 to 70 years, from various ethnic groups, geographical locations, and lifestyles^42^. The cohort sampling was performed using a mixed approach of voluntary participants (through advertisement and publicity campaigns) and cluster sampling. Written informed consent was taken for: (i) the study interview; (ii) the biophysical examination, (iii) blood taking, baseline blood tests and storage of bio-specimens; and (iv) future research and recontact. The inclusion criteria included being a Malaysian citizen, in possession of a valid identification card, not suffering from any acute illness at the time of study and giving informed consent to the study. Detailed information about each participant was collected along with blood and urine samples prior of eight hours fasting.

The sample size for this seroprevalence study was calculated using a single proportion formula for estimation of prevalence incorporated with Neyman allocation for stratified sampling based on estimation of 2.5% prevalence of hepatitis C in Malaysia^9^. Hence, a total of 1458 serum samples were randomly identified from the initial population of 106,527 (Supplementary Table S1). Sociodemographic data including age, gender, ethnicity, marital status, education levels, and history of illness were retrieved from TMC database. All samples were anonymized prior to analysis.

### Serological testing for hepatitis B and C markers

Five millilitres of venous blood were collected from each participant into a dry tube and centrifuged at 3000 rpm, for 10 min at 4 °C to separate the serum. The serum samples were aliquoted into cryotubes containing 500 µl each and stored at – 80 °C prior to use. Presence of hepatitis B surface antigen (HBsAg), antibody to hepatitis B core antigen (anti-HBc), and antibody to hepatitis C virus (anti-HCV) in the serum samples were screened by a chemiluminescence immunoassay (Roche Diagnostic, Germany; Abbott immunoassay, USA; Roche Diagnostic, Germany) on the Cobas analyser^43–45^. The results were interpreted in accordance with the manufacturers’ instructions. All equivocal results were retested using a sample from another cryotube belonging to the same individual. Samples reactive in the HCV screening assay were further tested to determine HCV RNA viral load and genotyping by Cepheid Xpert HCV Viral Load assay and sequencing, respectively^46,47^. Detection of HBsAg was considered indicative of chronic HBV infection and detection of anti-HCV was considered a marker of HCV infection^23^.

### Questionnaire-derived variables

Baseline information related to demographic and medical histories were collected using questionnaires and interviews^42^. Family history of hepatitis was determined by asking the participants if their biological parents or siblings were ever diagnosed with hepatitis. Participants were also asked about history of immunisation, chronic hepatitis, blood transfusion and surgery. Locality was defined as rural or urban for each participant.

### Missing data handling

Multiple imputation was performed by chained equations (MICE) with 25 cycles, based on a missing at random (MAR) assumption^48^. In each cycle, missing values for each variable were imputed based on a predictive distribution derived from regression on all other variables in the imputation model (education level, marital status, occupation, history of blood transfusion, chronic hepatitis and surgery, family history of hepatitis). Parameter estimates from the imputed datasets were combined using Rubin’s rules^49^.

### Ethics statement

Ethics approval was obtained from the institutional review and ethics board of Universiti Kebangsaan Malaysia (Project Code: FF-205-2007) in accordance with the Declaration of Helsinki. All participants gave their written consent before being recruited in this study.

### Statistical analysis

The overall prevalence of HBsAg, anti-HBc, and anti-HCV was expressed as the percentage of seropositive samples. The characteristics of the participants were compared with the serostatus of HBsAg, anti-HBc, and anti-HCV using chi-squared test. For statistical analysis, equivocal results were analysed together with negative results^50,51^. Multivariable logistic regression model was used to investigate associations of putative risk factors with seropositivity of HBsAg, anti-HBc, and anti-HCV adjusting for other confounding factors. For each analysis a variable selection process was used as previously described^52^. This involved an initially fitted a multivariable model including all selected risk factors, and then individually removing the least significant risk factor (p > 0.20), provided the likelihood ratio p-value for the nested models exceeded 0.20 and the estimated coefficients (on the logit scale) of all remaining variables did not differ by more than about 10%. Parameter estimates were expressed as odds ratios (OR) with 95% confidence interval (CI). A threshold of 0.05 was used for declaring significance. The risk explained by the classical risk factors was estimated using McFadden’s pseudo R^2^. All analyses were performed using STATA 14 (Stata Corporation, College Station, Texas, USA) and GraphPad Prism 7 (GraphPad Software Inc., USA).

## Supplementary information

Supplementary Information.
