# Supplementary material for: Seroprevalence of hepatitis B virus and hepatitis C virus infection among Malaysian population
Source: Sci Rep. 2020 Dec 3;10:21009. doi: 10.1038/s41598-020-77813-5 (PMC7713078; doi:10.1038/s41598-020-77813-5)
Supplement: Supplementary file 1 — Supplementary Information. [file 41598_2020_77813_MOESM1_ESM.docx]

**Seroprevalence of** **Hepatitis B virus and Hepatitis C virus infection among Malaysian population**

Nor Asiah Muhamad^1,2^, Rimah Melati Ab. Ghani^1,2^, Mohd Hatta Abdul Mutalip^2^, Eida Nurhadzira Muhammad^2^, Hasmah Mohamad Haris^2^, Rozainanee Mohd Zain^3^, Noraidatulakma Abdullah^4^, Nor Azila Muhammad Azami^4^, Nazihah Abd Jalal^4^, Norliza Ismail^4^, Nurul Ain Mhd Yusuf^4^, Raihannah Othman^4^, Azwa Shawani Kamalul Arifin^4^, Mohd Shaharom Abdullah^4^, Mohd Arman Kamaruddin^4^, Muhammad Radzi Abu Hassan^5,*^, Tahir Aris^2,3^, & Rahman Jamal^4,*^

^1^Evidence-based Healthcare Sector, National Institutes of Health, Ministry of Health Malaysia, 40170, Shah Alam, Malaysia;

^2^Center for Communicable Diseases Epidemiology Research, Institute for Public Health, National Institutes of Health, Ministry of Health Malaysia, 40170, Shah Alam, Malaysia;

^3^Institute for Medical Research, National Institutes of Health, Ministry of Health Malaysia, 40170, Shah Alam, Malaysia;

^4^ UKM Medical Molecular Biology Institute (UMBI), Universiti Kebangsaan Malaysia, Kuala Lumpur, Malaysia

^5^Hospital Sultanah Bahiyah, 05460, Alor Setar Kedah;

*Corresponding authors:

Tel: +603-91710415/+603-91459261;

Email: rahmanj@ppukm.ukm.edu.my; drradzi91@yahoo.co.uk

^*^share equal responsibility as supervisory personnel

Running head: Hepatitis B and C in Malaysia

**Supplementary**

**Supplementary Table 1**: The Malaysian Cohort’s participants characteristic (n=1458).

| **Characteristics** | **n (%)** | **95% CI** |
| --- | --- | --- |
| Year of recruitment |  |  |
| 2007 | 28 (2) | 1.2-2.7 |
| 2008 | 161 (11) | 9.5-12.6 |
| 2009 | 273 (19) | 16.7-20.8 |
| 2010 | 308 (21) | 19.1-23.2 |
| 2011 | 351 (24) | 21.8-26.3 |
| 2012 | 337 (23) | 21.1-25.3 |
|  |  |  |
| Age group |  |  |
| ≤44 | 399 (27) | 25.2-29.8 |
| 45-54 | 624 (43) | 40.3-45.4 |
| 55-64 | 415 (28) | 26.1-30.8 |
| 65-70 | 20 (1) | 0.8-1.9 |
|  |  |  |
| Gender |  |  |
| Male | 718 (49) | 46.5-51.6 |
| Female | 740 (51) | 48.4-53.5 |
|  |  |  |
| Ethnicity |  |  |
| Malay | 588 (40) | 37.9-42.8 |
| Chinese | 513 (35) | 32.6-37.8 |
| Indians | 180 (12) | 10.6-14.1 |
| Bumiputera Sabah | 93 (6) | 5.2-7.7 |
| Bumiputera Sarawak | 53 (4) | 2.7-4.7 |
| Others | 31 (2) | 1.4-2.9 |
|  |  |  |
| Marital status |  |  |
| Single | 73 (5) | 4.0-6.3 |
| Married | 1300 (89) | 87.5-90.7 |
| Widow/Widower | 62 (4) | 3.3-5.4 |
| Separated | 5 (0) | 0.1-0.8 |
| Divorced | 18 (1) | 0.8-2.0 |
|  |  |  |
| Education level |  |  |
| No formal education | 25 (1) | 1.1-2.5 |
| Primary | 417 (29) | 26.3-31.0 |
| Secondary | 742 (51) | 48.3-53.4 |
| Tertiary | 274 (19) | 16.9-20.9 |
|  |  |  |
| Occupation |  |  |
| Unemployed | 500 (34) | 31.9-36.8 |
| Non-Government | 628 (43) | 40.5-45.6 |
| Government | 248 (17) | 15.2-19.0 |
| Self-employed | 82 (5) | 4.6-6.9 |
|  |  |  |
| State |  |  |
| Johor | 132 (9) | 7.5-10.7 |
| Kedah | 68 (5) | 3.6-5.8 |
| Kelantan | 48 (3) | 2.4-4.3 |
| Melaka | 19 (1) | 0.8-1.9 |
| Negeri Sembilan | 58 (4) | 3.0-5.1 |
| Pahang | 74 (5) | 4.0-6.3 |
| Perak | 122 (8) | 7.0-9.9 |
| Pulau Pinang | 48 (3) | 2.3-4.2 |
| Sabah | 145 (10) | 8.5-11.5 |
| Sarawak | 113 (8) | 6.4-9.1 |
| Selangor | 343 (23) | 21.3-25.9 |
| Terengganu | 40 (3) |  |
| WP Kuala Lumpur | 245 (17) | 14.9-18.7 |
| WP Putrajaya | 3 (0) | 0.0-0.5 |
|  |  |  |
| Locality |  |  |
| Urban | 1035 (71) | 68.8-73.4 |
| Rural | 423 (29) | 26.6-31.2 |
|  |  |  |
| Immunisation history |  |  |
| No | 1198 (82) | 80.1-84.1 |
| Hepatitis A | 41 (3) | 2.1-3.8 |
| Hepatitis B | 180 (12) | 10.8-14.1 |
| Hepatitis A & Hepatitis B | 39 (3) | 1.8-3.5 |
|  |  |  |
| History of chronic hepatitis disease |  |  |
| Yes | 2 (1) | 0.0-0.5 |
| No | 1456 (99) | 99.5-99.9 |
|  |  |  |
| Family history of Hepatitis |  |  |
| Yes | 0 (0) | 0 |
| No | 1458 (100) | 100 |
|  |  |  |
| History of surgery |  |  |
| Yes | 794 (54) | 51.9-57.0 |
| No | 664 (46) | 43.0-48.1 |
|  |  |  |
| History of blood transfusion |  |  |
| Yes | 117 (8) | 6.7-9.5 |
| No | 1341 (92) | 90.5-93.3 |

* indicate significant differences, p<0.05
